# Pronounced Neuroplasticity in the Primary Visual Cortex of the 13-Lined Ground Squirrel during Hibernation

**DOI:** 10.1523/JNEUROSCI.0077-26.2026

**Published:** 2026-05-18

**Authors:** Allison Fultz, Carlos A. Mejias-Aponte, Christina Jacob, Laura Castillo, Francisco M. Nadal-Nicolas, Yue Gao, Wei Li, Hendrikje Nienborg

**Affiliations:** ^1^Laboratory of Sensorimotor Research at the National Eye Institute, Bethesda, Maryland 20892; ^2^Retinal Neurophysiology Section at the National Eye Institute, Bethesda, Maryland 20892

**Keywords:** 13-lined ground squirrel, dendrites, hibernation, morphology, neuroplasticity, primary visual cortex, pyramidal neuron, spiny stellate neuron

## Abstract

Hibernating animals can show neuroplasticity throughout the hibernation season. In ground squirrels, decreased dendritic arborization in the hippocampus, somatosensory cortex, and thalamus during deep hibernation (“torpor”) suggests that this neuroplasticity is a brain-wide phenomenon. However, the degree to which neuroplasticity occurs in the visual system is not clear. While transient retinal changes have been reported during torpor, neuroplasticity beyond the retina remains unknown. Here, we characterized hibernation-related neuroplasticity in the primary visual cortex (V1), the first cortical area to receive visual information, in the 13-lined ground squirrel (*Ictidomys tridecemlineatus*). We compared neuronal morphology in Golgi-stained samples from male and female hibernating or nonhibernating squirrels. For the hibernating squirrels, the brain tissue was sampled during two different epochs: torpor and intertorpor arousal. Dendritic arborization decreased during torpor in V1 layer 2/3 pyramidal neurons, manifesting as decreases in dendritic length, number, and complexity. These changes fully reversed during intertorpor arousal, indicating that on average dendritic arbors grew by 0.75 mm (65%) over ∼1.5 h. No morphological differences between hibernating and nonhibernating squirrels were apparent when compared 6 months after the hibernation season. We also found no neuroplastic changes in V1 layer 4 spiny stellate neurons, unlike in this cell type in the somatosensory cortex. Together, this revealed, for the first time, hibernation-related neuroplasticity in V1 in support of a brain-wide mechanism but with area-specific differences. The speed and magnitude of this naturally occurring neuroplasticity could make ground squirrel V1 a powerful translational model system for conditions requiring neuroplasticity, such as recovery from stroke.

## Significance Statement

This study is the first demonstration of pronounced hibernation-related neuroplasticity in the primary visual cortex of ground squirrels. Layer 2/3 pyramidal neurons in the primary visual cortex (V1) reduced arborization during torpor. Within 1.5 h after arousal from torpor, the arborization reversed to nonhibernation levels. The extent and speed of this naturally occurring neuroplasticity could make the relatively well-understood V1 of ground squirrels a powerful translational model system. Complementing insights on neuroplasticity in V1 during development, it has the potential to be leveraged for the study of treatment mechanisms and conditions requiring neuroplasticity, ranging from neurodegeneration to recovery after stroke.

## Introduction

Squirrels are diurnal, highly visual mammals (Van Hooser and Nelson, 2006). Their cone-dominated retina provides a research model for cone physiology and recovery from injury (Linberg et al., 2002; Li and DeVries, 2004; Merriman et al., 2016). As a highly visual rodent, squirrels have larger visual brain areas than nocturnal rodents, including the primary visual cortex (V1; Lane et al., 1971; Kaas and Collins, 2001; Campi and Krubitzer, 2010; Xiao et al., 2021). Throughout their lives, ground squirrels experience scarce food resources and exposure to cold temperatures, which results in drastic metabolic challenges that they survive by hibernating (Li, 2020), but apart from studies in the retina (Kuwabara, 1975; Reme and Young, 1977; Luan et al., 2018; Sajdak et al., 2018; Ball et al., 2022; Ozbek et al., 2026), little is known about how hibernation affects the visual system.

**Figure 1.**
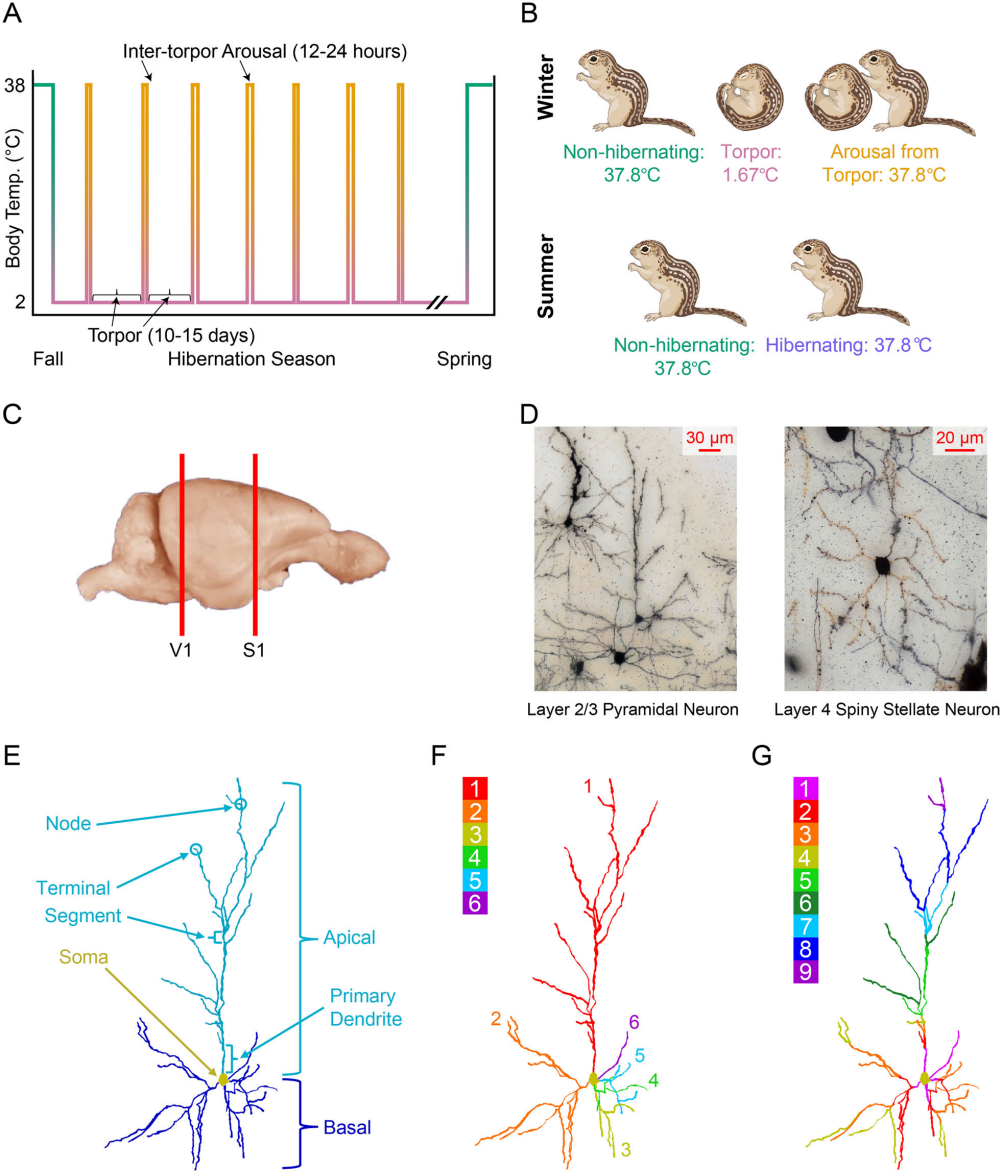
Experimental design. ***A***, Schematic of body temperature variation at the different epochs of the hibernation season. Awake (38°C), torpor (2°C), and intertorpor arousal (38°C) epochs are labeled in their corresponding colors for the conditions in the winter group: nonhibernating (green), torpor (pink), and arousal from torpor (orange). ***B***, Squirrels in the winter and summer groups, categorized into conditions representing the natural epochs of hibernation. In the winter group, there were three conditions: nonhibernating (green, *N* = 4), torpor (pink, *N* = 4), and arousal from torpor (orange, *N* = 4). In the summer group, there were two conditions: nonhibernating (green, *N* = 4) and hibernating (purple, *N* = 4). ***C***, Atlas locations of V1 and S1 on a sagittal view of the 13-lined ground squirrel brain (Kaas et al., 1972; Wong and Kaas, 2008). ***D***, Example V1 layer 2/3 pyramidal neuron (left) and V1 layer 4 spiny stellate neuron (scaled at 20 µm; right, note the different scale bar) depicting the differences in morphology. We captured images using 3D bright-field volumes microscopy at 40× magnification. ***E***, V1 layer 2/3 pyramidal neuron trace with labeled apical dendrite (blue), basal dendrites (navy), and soma (yellow). We also marked the variables used for analysis in Neurolucida Explorer: node (branching point), terminal (endpoint), soma (cell body), primary dendrite (portion of the dendrite extending from the soma), and segment (portion of the dendrite between two nodes). ***F***, V1 layer 2/3 pyramidal neuron trace with labeled dendrites. Dendrite number is labeled by color. ***G***, V1 layer 2/3 pyramidal neuron trace colored by ascending branch order.

Hibernation in ground squirrels consists of several phases (Fig. 1*A*) that are controlled by specialized circuits in the hypothalamus. Previous work implicated the suprachiasmatic nucleus (Kilduff et al., 1982; Kilduff et al., 1990; Bratincsak et al., 2007), and recent work identified a specific hypothalamic circuit to induce artificial torpor in mice (Harting and Huerta, 1983; Hrvatin et al., 2020; Takahashi et al., 2020). In ground squirrels, during the phase of deep hibernation, torpor, metabolic activity is reduced, and the animals' body temperature drops to 2–3°C above the surrounding environment (Mohr et al., 2020). In the 13-lined ground squirrel, torpor epochs last 7–10 d and are interrupted by bouts of arousal (interbout arousal) lasting ∼1 d, when metabolic activity increases and body temperature returns to euthermia (Muleme et al., 2006; Andrews, 2019). Morphological studies of the hippocampus, somatosensory thalamus, and primary somatosensory cortex (S1) in ground squirrels have shown a significant reduction of the dendritic arbor during torpor that reverses during the interbout arousal phases (Popov et al., 1992; von der Ohe et al., 2006). However, it is unknown whether V1 exhibits similar changes as the somatosensory system throughout hibernation or whether in these highly visual rodents, the effects of hibernation in V1 are distinct.

Here, we investigated the effect of hibernation on the dendritic morphology of V1 neurons. To compare our findings with earlier work, we focused on two types of excitatory neurons in V1, pyramidal and spiny stellate neurons. We collected brain samples in the spring from squirrels in torpor, aroused from torpor, and squirrels that did not go through hibernation. To identify long-lasting changes in the morphology of V1 neurons, we additionally collected brains in the summer from squirrels that either had or had not hibernated in the preceding winter. To compare effects across sensory modalities, we also examined S1 pyramidal and spiny stellate neurons. We found that dendritic arbors shrink during torpor and regrow back within 1.5 h during the arousal from torpor in V1 and S1 layer 2/3 pyramidal neurons, as well as in S1 layer 4 spiny stellate neurons. Conversely, V1 layer 4 spiny stellate dendritic arbors did not show systematic changes with torpor. Together, our work identified pronounced torpor-related plasticity in V1 neurons as well as differences across cortical areas and cell types.

## Materials and Methods

### Animals

We purchased male and female 13-lined ground squirrels (*Ictidomys tridecemlineatus*) from the University of Wisconsin in Oshkosh (Merriman et al., 2012; Ou et al., 2018). When the experiments were conducted, the squirrels' ages ranged from 9 to 15 months. Upon arrival, we subcutaneously implanted each squirrel with an identification and temperature recorder in the midscapular area (1.4 × 0.2 cm, BMDS IPTT-300, Bio Medic Data Systems). Each squirrel was housed individually in cages (9.5ʺ × 13.5ʺ × 8.5ʺ), room temperature was set to ∼20–24°C and 30–70% humidity. To simulate the squirrels’ natural habitat lighting, the lights-on period was set every 2 weeks to sunrise and sundown times in Wisconsin.

To better monitor hibernation phases, we implanted wireless temperature bio-loggers (2.5 × 0.8 cm, DST micro-ACT, Star-Oddi) subcutaneously in the area posterior to the subscapular area under isoflurane anesthesia. Following an incision in the subscapular area, a subcutaneous pocket was created, and the transponder was inserted ∼1–2 cm away from the incision site. This helps reduce tension on the sutures and prevents the wound from reopening. The bio-logger recorded body temperature every 15 min, and the data collected were retrieved via telemetry. To induce torpor, we lowered the temperature of the vivarium to 4°C and food-restricted the squirrels. We moved the squirrels into the hibernaculum in their homecages after signs of decreased physiological activity, including ataxia, unresponsiveness, reduced water intake, and decreased waste secretion, were observed. In the hibernaculum, the temperature was set to 4°C with no light and 30–70% humidity. During monitor visits, we used a red light. After 3 d of monitoring for signs of torpor, squirrels were transferred into 4.4 quart Handi-Boxes (Handi-Box) with 0.5 inch Aspen shave bedding with holes for ventilation. To gauge activity during intertorpor arousal, we placed sunflower seeds in discernible locations and monitored any shifts in their placements. No water nor environmental enrichment was provided. All procedures were approved by the Institutional Care and Use Committee of the National Eye Institute and followed the Animal Welfare Act (NIH/DHHS), under the animal study protocol NEI-595.

### Experimental design

We assigned two male and two female 13-lined ground squirrels to each of five experimental conditions. Three of the experimental groups' brains were collected in the winter, within the hibernation season, and the other two in the summer. The squirrels in the winter groups were 9–10 months old, and those in the summer groups were 15 months old. The winter experimental groups were (1) nonhibernating, (2) torpor, and (3) arousal from torpor. In the nonhibernating condition, squirrels did not go through hibernation and stayed out of the hibernaculum under standard housing described above; we collected their brains in the morning when they were active and awake. In the torpor condition, subjects were brought out of the hibernaculum in the Handi-Box while in deep torpor, and their brains were collected immediately. In the arousal from torpor condition, we aimed to mimic the interbout arousal within the hibernation cycle (Grabek et al., 2019; Fig. 1*A*,*B*). The squirrels in the arousal from torpor condition were taken out of the hibernaculum in their Handi-Box while in deep torpor. In our procedure room, the squirrels were transferred to standard cages and placed under a white light of 300–500 lx at room temperature. The brains of the subjects in the arousal from torpor condition were collected when the body temperature reached euthermia (∼37°C, body temperature of awake subjects) within 1–1.5 h after being taken out of the hibernaculum. The summer experimental groups were (1) nonhibernating winter and (2) hibernating winter. Like the nonhibernating condition in the winter cohort, the squirrels in the nonhibernating group had not experienced hibernation in the previous hibernation season, while the squirrels in the hibernating group had gone through hibernation during the preceding winter (Fig. 1*A*,*B*). We collected the brains of the two summer groups in the morning when the squirrels were active and awake.

### Tissue collection

We deeply anesthetized the squirrels via exposure to 5% isoflurane in a 30 L glass anesthesia induction chamber kept inside a fume hood. We monitored the squirrel for lack of movement and breathing for at least 2 min; when no eye reflex and motor response to paw pinch were present, the eyes were resected for a separate experiment. We then exsanguinate the squirrels via intracardiac perfusion with 200 ml of 0.9% saline. We extracted the brain and, with a razor blade, cut sagittally along the midline. We cut the right hemispheres into anterior, medial, and posterior blocks and transferred them into 20 ml vials containing 15 ml of Golgi–Cox solution (Zaqout and Kaindl, 2016). The left hemisphere was transferred to 4% paraformaldehyde, 1% acrylamide, and 0.25% bis-acrylamide for 48 h at 4°C, before a 3-h-long incubation at 37°C to preserve the sample within a hydrogel (Chung et al., 2013).

### Histology

The brain blocks collected into Golgi–Cox solution were kept overnight in the dark at room temperature. On Day 2, each block was transferred to another 20 ml vial containing 15 ml of Golgi–Cox solution, returned to the dark, and kept at room temperature for 14 d. The Golgi–Cox solution consisted of a mix of 1.04% potassium dichromate, 1.04% mercury chloride, and 0.83% potassium chloride (Zaqout and Kaindl, 2016). We transferred the samples into a cryoprotectant solution containing 30% sucrose, 30% ethylene glycol, and 0.1% polyvinylpyrrolidone in 0.05 M phosphate buffer saline. We transferred the samples into fresh cryoprotectant solution every other day for 2–5 d until sectioning. We cut coronal 100 µm sections from each tissue block using a vibratome (Campden Instruments, 7000 smz-2). The tissue was collected into 12-well plates containing the cyroprotectant solutions and stored in the fridge until mounting. For the winter group, we blot-mounted slices on gelatin-subbed slides. For the summer group, we blot-mounted slices on positively charged slides and dried them on a tissue warmer at ∼4.4°C for two hours. We dried and stored all slides in the dark for 2–3 d at room temperature before developing the Golgi–Cox reaction. Using established protocols described by Zaqout and Kaindl (2016), we developed the Golgi–Cox staining by transferring the slides through baths of 50% ethanol, 3:1 ammonia/dH_2_O, and 5% sodium thiosulfate before dehydration with ethanol (70, 95, and 100%) and xylene substitute. Using Eukitt mounting media (Electron Microscopy Sciences), we coverslipped the slides and cured them using UV light for at least 30 s.

### Imaging

We identify the squirrels’ primary visual and somatosensory cortex using previously published anatomical references (Kaas et al., 1972; Wong and Kaas, 2008). We used the shape of the hippocampus, the striatum, and the thalamus across the anterior–posterior axis to locate our anatomical targets. Bright-field images were acquired using an Olympus VS200 or a Zeiss Axio Imager.M2. We collected 3D volumes with an air 40× objective with a 0.95 numerical aperture, with a voxel size of 0.1384, 0.1384, and 0.84 µm for *X*, *Y*, and *Z* planes, respectively. For the winter group, we imaged 60 S1 layer 4 spiny stellate neurons (5 per squirrel, 20 per condition) and 90 S1 layer 2/3 pyramidal neurons (5 from two squirrels and 10 from two squirrels in each condition). We imaged 60 V1 layer 4 spiny stellate neurons (5 per squirrel and 20 per condition except in the torpor condition, for which 7 were collected from two squirrels and 6 from one squirrel due to tissue loss) and 60 V1 layer 2/3 pyramidal neurons (5 per squirrel, 20 per condition). For the summer group, we imaged 40 V1 layer 2/3 pyramidal neurons (5 per squirrel and 20 per condition, except in the hibernating condition, for which 7 were collected from two squirrels and 6 from one squirrel). To select neurons, we made sure that all dendrites were complete and visible with blunt ends, suggesting no early termination due to tissue slicing. Pyramidal neurons were selected for their thicker, longer apical dendrites, basal dendrites, and triangular somas, while spiny stellate cells were selected for their rounder somas, radial symmetry, and obvious dendritic spines (Fig. 1*D*).

### Morphological measures

We hand-traced all neurons in Neurolucida 360 (MicroBrightField). For the winter group, tracers were blind to experimental conditions. For each layer 2/3 pyramidal neuron, we traced the soma, the apical dendrite, and the basal dendrites (see example neurons in Fig. 1*E*). For each layer 4 spiny stellate neuron, we traced the soma and dendrites. For each neuron, we included the following measures: (1) soma surface area (square micrometers); (2) number of dendrites, i.e., the number of dendritic processes emerging from the soma (Fig. 1*F*); (3) dendrite length (micrometer); (4) complexity [(sum of terminal orders + number of terminals) × (total dendrite length/number of primary dendrites)]; and (5) number of nodes. We also ran a branch order analysis to evaluate the number of segments (portions of a dendrite between two branching points) at each branching (see example in Fig. 1*G*). In the branch order analysis, the segment that begins at the origin of a dendrite is assigned Branch Order 1. Therefore, for each neuron, the number of primary dendrites equals the number of segments with Branch Number 1. Additionally, we ran a Sholl branching analysis, generating 3D spheres from the center of the soma at 10 µm increments (Sholl, 1953). From the Sholl analysis, we measured the number of dendritic intersections with each shell boundary and the total dendritic length (micrometer) within each sphere.

### Data analysis

We conducted statistical analyses separately for each group (winter or summer), neuron type (layer 2/3 pyramidal or spiny stellate), and brain regions (V1 or S1). For pyramidal neurons, we analyzed basal, apical, and total dendrites separately, and for spiny stellate neurons, we analyzed total dendrites. For branch order analysis, we calculated the segment number up to Branch Order 7 and Sholl up to 120 µm from soma for pyramidal neurons and 90 µm from soma for spiny stellate neurons.

For visualization in Figures 3–5, we show neurons with the minimum and maximum complexity values in the first and last quintile, respectively. For the middle quintiles, we chose neurons with median complexity values within those quintiles.

We computed all statistical tests using GraphPad Prism version 10.2.0 (GraphPad Software) with an alpha value of 0.05. We tested normality using the D’Agostino–Pearson’s test and equal variance using Bartlett's test, when comparing more than two groups and or the *F* test when comparing two groups. Because most of our data did not meet the conditions of normality and/or equal variances, we reported all the results using nonparametric tests. When comparing more than two groups, we used the Kruskal–Wallis test with Dunn's test for multiple comparisons. The *p* values reported here are adjusted *p* values. When comparing two groups, we used the Mann–Whitney test. To ease visually comparing groups, we reported means and standard error of the mean.

### Data availability

The data supporting the findings reporting in this study will be made available from the corresponding author upon reasonable request.

## Results

Our goal was to determine how the metabolic and temperature stressors of hibernation affect V1 neurons in 13-lined ground squirrels. We hypothesized that hibernation would cause transient morphological changes since such transient changes have been identified upstream, in the retina of ground squirrels (Kuwabara, 1975). Guided by findings in other brain areas (von der Ohe et al., 2006), we examined plasticity in dendrite morphology. We collected and Golgi-stained brain samples from ground squirrels during different hibernation phases (Fig. 1*A*). At the end of the hibernation season in March (winter groups), we collected the brain tissue from three groups of squirrels (Fig. 1*A*,*B*). The control group was squirrels that had been prevented from hibernating during the hibernation season (nonhibernating). The second group of squirrels was in deep torpor (torpor). The third group of squirrels was aroused from torpor, and brain samples were collected ∼1.5 h later, when the squirrels' body temperature had reached euthermic values (arousal from torpor). To probe for long-term changes, we collected additional samples in the summer following the hibernation season (summer groups). The two summer groups were squirrels that had or had not hibernated in the preceding winter (Fig. 1*B*, bottom). Throughout, we primarily focused on one major excitatory neuron type in rodent V1, layer 2/3 pyramidal neurons (Stratford et al., 1996; Helmstaedter et al., 2008; Weis et al., 2025) and compared it with somatosensory (S1) layer 2/3 pyramidal neurons and layer 4 spiny stellate cells in V1 and S1.

### V1 layer 2/3 pyramidal neurons’ dendritic arborization is reduced during torpor, and this reduction reverses during the arousal from torpor

**Figure 2.**
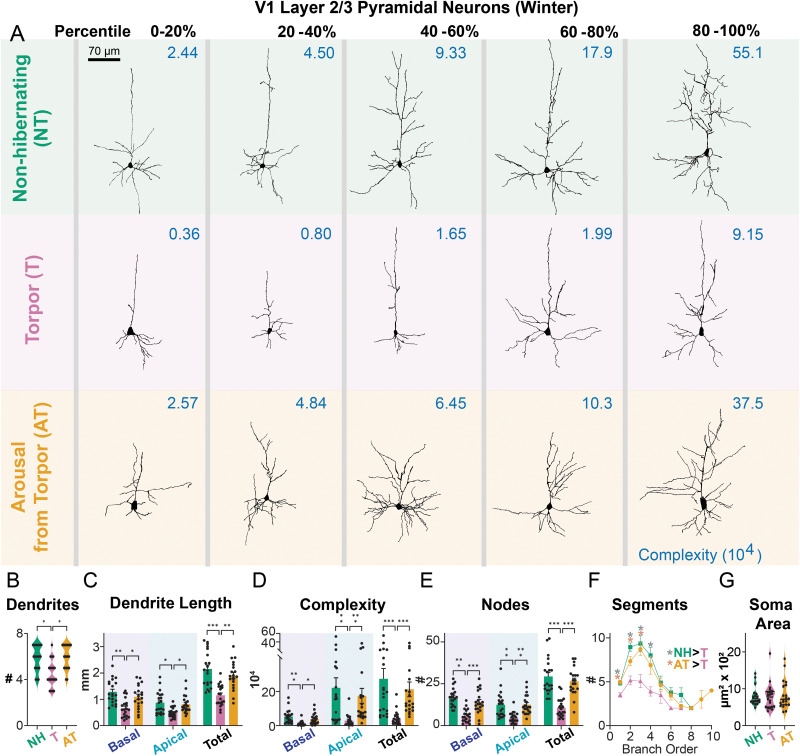
Dendritic arborization in V1 layer 2/3 pyramidal neurons is reduced during torpor. ***A***, For each condition (rows), neurons are ranked by complexity (blue, in 10^4^) and example neurons shown for each quintile. Note the sparser dendritic arbors for the torpor group. ***B***, Neurons had fewer dendrites in the torpor condition (mean = 4.5) compared with the nonhibernating (mean = 5.9; *p* < 0.01) and arousal from torpor (mean = 6.0; *p* = 0.001) conditions. ***C***, Basal and apical dendrite length were shorter in the torpor (basal mean = 0.7 mm; apical mean = 0.5 mm) condition compared with the nonhibernating (basal mean = 1.3 mm; apical mean = 0.9 mm) condition (basal, *p* < 10^−3^; apical, *p* < 0.01) and the arousal from torpor (basal mean = 1.1 mm; apical mean = 0.8 mm) condition (both, *p* < 0.01). ***D***, Complexity was lower in the torpor (basal mean = 6.4k; apical mean = 15k) condition compared with the nonhibernating (basal mean = 27k; *p* < 10^−4^; apical mean = 111k; *p* < 10^−3^) and arousal from torpor conditions (basal mean = 19k; *p* < 0.01; apical mean = 87k; *p* < 10^−4^). ***E***, There were fewer nodes in the torpor (basal mean = 5.9; apical mean = 4.9) condition compared with the nonhibernating (basal mean = 16.9; *p* < 10^−4^; apical mean = 12.5; *p* < 10^−3^) and arousal from torpor (basal mean = 14.0; *p* < 10^−3^; apical mean = 12.5; *p* < 10^−4^) conditions. ***F***, Neurons also had fewer basal segments in the torpor condition compared with the nonhibernating condition at Branch Orders 1–4 and compared with the arousal from torpor condition at Branch Orders 1–3. ***G***, No significant differences in soma surface area across conditions. *p < 0.01; **p < 10^−3^; ***p < 10^−4^.

We observed a reduction in dendritic arborization of V1 layer 2/3 pyramidal neurons during torpor that reversed during arousal from torpor (Fig. 2). To compare dendritic morphology across the hibernation season, we organized the traces of the neurons in each condition (rows in Fig. 2*A*) by ascending complexity, a metric that combines dendrite length and branching. We divided the neurons in each condition into quintiles and show one representative neuron for each quintile in Figure 2*A*. When comparing neuronal morphology across conditions, systematic differences in arborization were visually apparent. Neurons in the torpor condition (second row) had less dendritic arborization in each quintile compared with neurons in the nonhibernating condition (first row). This reduction in dendritic arborization reversed for neurons in the arousal from torpor condition (third row) to comparable levels to the neurons in the nonhibernating group.

Our quantitative analysis of the neuron morphology also supports the reduction of dendritic arborization in V1 layer 2/3 pyramidal neurons during torpor, as well as a reversal within 1.5 h of arousal from torpor. The decreased arborization during torpor resulted from a smaller number of dendrites (Fig. 2*B*), reduced dendrite length (Fig. 2*C*), complexity (Fig. 2*D*), and fewer dendritic nodes (Fig. 2*E*). These differences were comparable and statistically significant for both basal and apical dendrites. When the measurements of the basal and the apical dendrites are combined, the number of nodes during torpor was decreased by 64% and dendrite length by 65% compared with the nonhibernating group. This reduction in arborization during torpor was more pronounced close to the soma, as shown in the branch order and Sholl analysis (Figs. 2*F*, 6*A*). In contrast, the soma size did not differ significantly across conditions.

Although the interval from torpor to arousal in the arousal-from-torpor groups was only ∼1.5 h, it was sufficient to reverse the reduction in dendrite arborization observed during torpor. The arborization levels mirror those of squirrels that did not hibernate, and consequently the number of dendrites, dendrite length, and dendrite nodes do not differ significantly between the arousal-from-torpor and nonhibernating groups (Fig. 2*C*–*F*). Summed over apical and basal dendrites, this corresponds to an average dendrite regrowth per neuron of 0.75 mm within 1.5 h (Fig. 2*C*, right). These findings suggest that the hibernation-related changes in arborization were pronounced and transient. To further test whether the hibernation-related changes fully reversed, we also compared the neuronal arborization in the summer in squirrels that had or had not hibernated in the preceding winter.

### Experiencing hibernation during winter does not produce differences in arborization of V1 layer 2/3 pyramidal neurons in the summer

**Figure 3.**
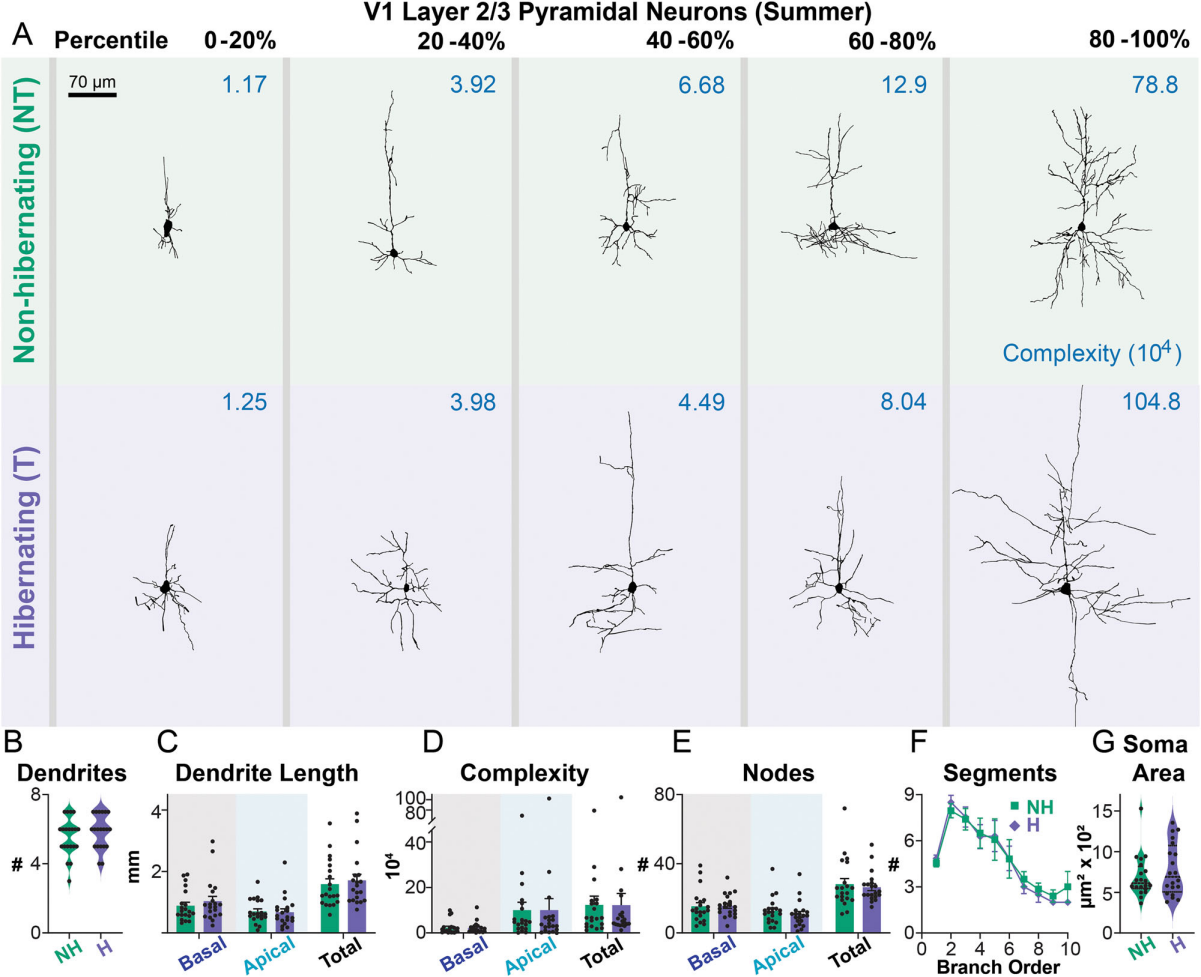
Dendritic arborization is comparable between V1 layer 2/3 pyramidal neurons 6 months after the hibernation season. ***A***, Format as in Figure 2*A* but for the summer groups. Note the similarity of arborization across conditions. There were no significant differences in the number of dendrites (***B***), dendrite length (***C***), complexity (***D***), number of nodes (***E***), basal segment number at any branch order (***F***), or soma surface area across conditions (***G***).

Contrasting with the changes during torpor, 6 months after the hibernation season, there were no significant differences in dendritic arborization of V1 layer 2/3 pyramidal neurons of squirrels that hibernated in the winter compared with those that had not hibernated. The arborization apparent in neuronal traces from the two groups of animals appear similar when visually inspected in ranked quintiles (Fig. 3*A*) based on complexity. This visual impression was confirmed by our statistical analysis. There were no significant differences in morphological measures across the groups, including the number of dendrites, dendritic length, number of nodes, and branch order (Fig. 3*B*–*E*). Similarly, in the Sholl analysis, there were no differences across the groups in the number of intersections or dendritic length with a Sholl shell (Fig. 6*C*). These results provide further evidence that torpor does not result in long-lasting changes of dendritic arborization in V1 layer 2/3 pyramidal neurons.

### Dendritic arborization in V1 layer 4 spiny stellate neurons remains unchanged during torpor

**Figure 4.**
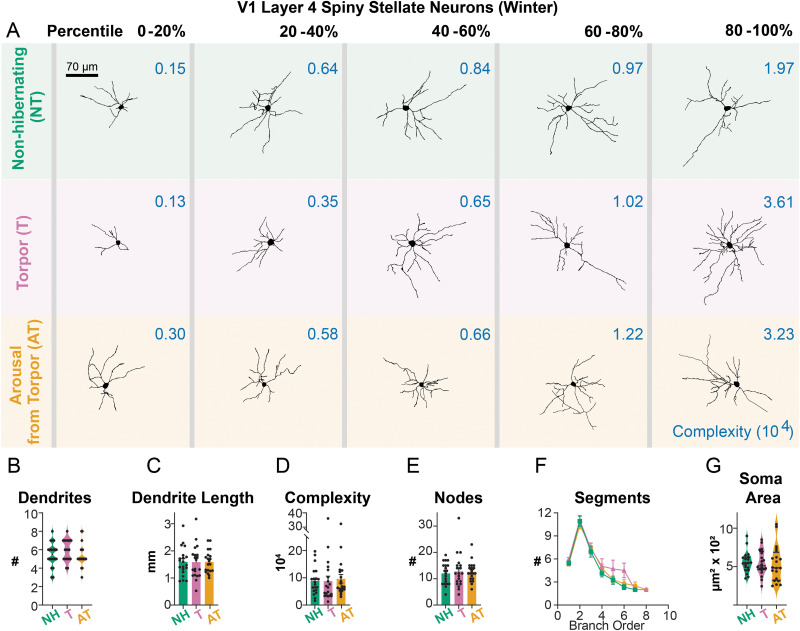
Dendritic arborization remains unchanged in V1 layer 4 spiny stellate neurons during torpor. ***A***, Format as in Figure 2*A* but for layer 4 spiny stellate neurons. The arborization was overall similar across conditions. There were no significant differences in the number of dendrites (***B***), dendrite length (***C***), complexity (***D***), or the number of nodes (***E***), basal segment number at any branch order (***F***) or soma surface area (***G***) across conditions.

A less common cell type in rodent V1, layer 4 spiny stellate neurons, which provides input to layer 2/3 pyramidal neurons, did not show torpor-related reduced or other hibernation-related changes in arborization. Dendritic arbors from neurons in the nonhibernating, torpor, and arousal from torpor groups appear similar when visually inspected in quintiles ranked by complexity (Fig. 4*A*). Moreover, we found no statistical differences between the groups in the number of dendrites, dendritic length, number of nodes, and branch order (Fig. 4*B*–*E*). Similarly, the Sholl analysis revealed no differences across the groups in the number of intersections or dendritic length as a function of Sholl shell (Fig. 6*B*). This absence of torpor-related changes in spiny stellate layer 4 V1 cells was surprising, since transient hibernation-related plasticity has previously been reported for spiny stellate layer 4 neurons in the somatosensory cortex S1 of ground squirrels (von der Ohe et al., 2007). However, unlike in V1, layer 4 spiny stellate neurons are a major cell type in the rodent somatosensory cortex (Schubert et al., 2003). We therefore sought to confirm the previous findings of hibernation-related changes in S1 layer 4 spiny stellate neurons in our study and additionally probe for such changes in S1 layer 2/3 pyramidal neurons.

### S1 layer 2/3 pyramidal and layer 4 spiny stellate neurons’ dendritic arborization is reduced during torpor while reversing its reduction during the arousal from torpor

**Figure 5.**
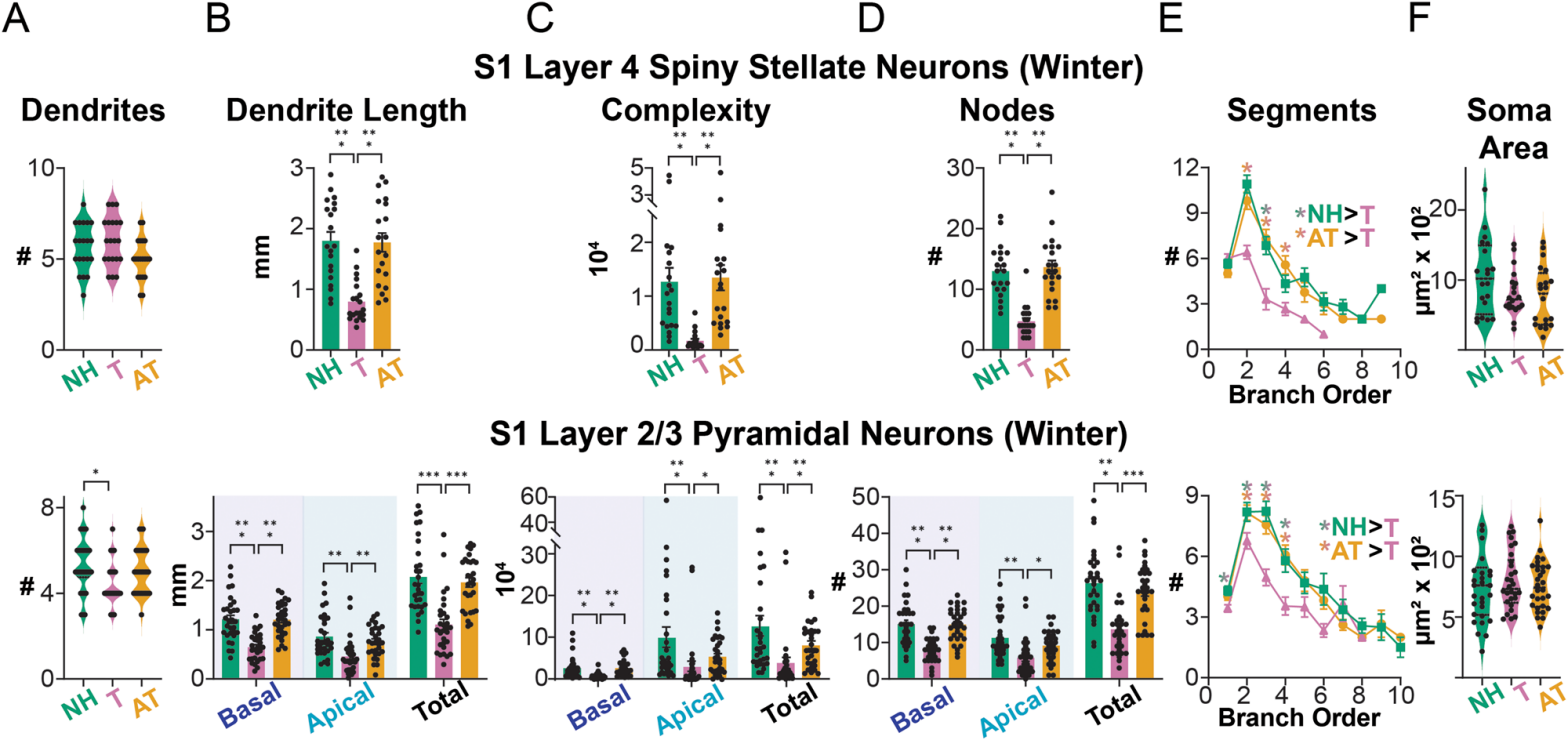
Dendritic arborization in S1 layer 4 spiny stellate and layer 2/3 pyramidal neurons is reduced during torpor. ***A–F***, Data for S1 layer 4 spiny stellate neurons. ***A***, There were no significant differences in dendrite number across conditions. ***B***, Dendrite length was shorter in the torpor (mean = 0.4 mm) condition compared with the nonhibernating (mean = 0.9 mm; *p* < 10^−4^) and arousal from torpor (mean = 0.9 mm; *p* < 10^−4^) conditions. ***C***, Complexity was lower in the torpor (mean = 1.7k) condition compared with the nonhibernating (mean = 13k; *p* < 10^−4^) and arousal from torpor (mean = 13k; *p* < 10^−4^) conditions. ***D***, There were fewer nodes in the torpor (mean = 4.7) condition compared with the nonhibernating (mean = 13.0; *p* < 10^−4^) and arousal from torpor (mean = 13.7; *p* < 10^−4^) conditions. ***E***, Segment number was lower in the torpor condition compared with the nonhibernating condition at Branch Orders 2–3 and compared with the arousal from torpor condition at Branch Orders 2–4. ***F***, There were no significant differences in the soma surface area across conditions. ***G–L***, Data for the S1 layer 2/3 pyramidal neurons. ***G***, Dendrite number was lower in the torpor (mean = 4.4) condition compared with the nonhibernating (mean = 5.3; *p* < 0.01) condition. ***H***, Basal and apical dendrite length were significantly shorter in the torpor (basal mean = 0.7 mm; apical mean = 0.5 mm) condition compared with the nonhibernating (basal mean = 1.2 mm; *p* < 10^−4^; apical mean = 0.9 mm; *p* = 10^−4^) and arousal from torpor (basal mean = 1.2 mm; *p* < 10^−4^; apical mean = 0.8 mm; *p* < 10^−3^) conditions. ***I***, Complexity was lower in the torpor (basal mean = 8k; apical mean = 30k) condition compared with the nonhibernating (basal mean = 27k; apical mean = 101k) condition (total: *p* < 10^−4^) and compared with the arousal from torpor (basal mean = 27k; apical mean = 55k) condition (basal, *p* < 10^−4^; apical, *p* < 0.01). ***J***, The number of nodes was lower during the torpor (basal mean = 7.8; apical mean = 5.8) condition compared with the nonhibernating (basal mean = 15.1; *p* < 10^−4^; apical mean = 11.3; *p* = 10^−4^) and arousal from torpor (basal mean = 14.9; *p* < 10^−4^; apical mean = 9.1; *p* < 0.01) conditions. ***K***, Basal segment number was lower during the torpor condition compared with the nonhibernating condition at Branch Orders 1–4 and compared with the arousal from torpor condition at Branch Orders 2–4. ***L***, There were no significant differences in the soma surface area across conditions. *p < 0.01; **p < 10^−3^; ***p < 10^−4^.

**Figure 6.**
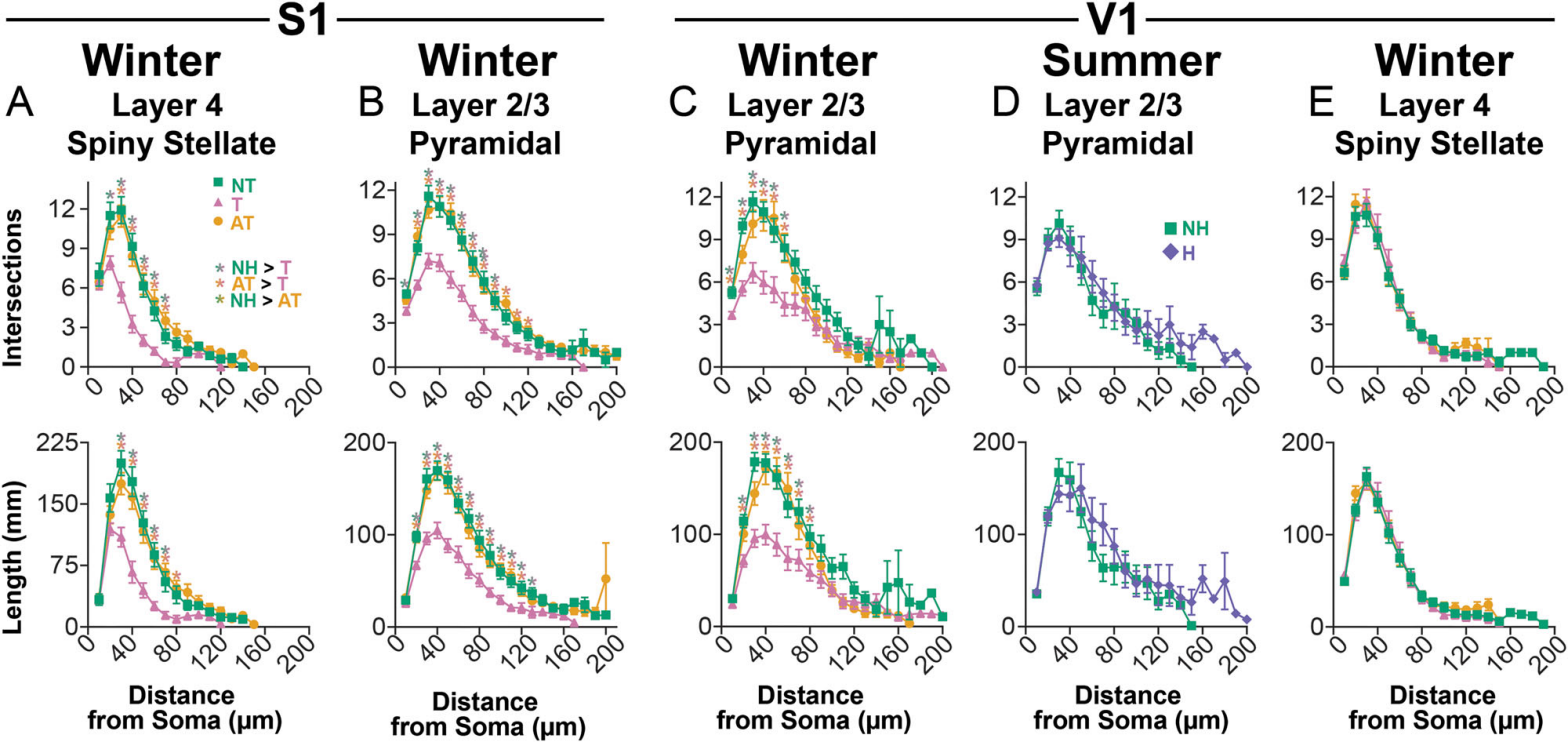
Reductions in dendritic arborization during torpor in S1 neurons and V1 layer 2/3 pyramidal neurons were more pronounced closer to the soma. ***A***, For S1 layer 4 spiny stellate neurons, there were fewer dendritic intersections in the torpor condition than in the nonhibernating condition between 20 and 70 μm and the arousal from torpor condition between 30 and 70 μm. Dendrite length was shorter in the torpor condition than in the nonhibernating and the arousal from torpor condition between 30 and 80 μm. ***B***, Same as ***A*** but for basal dendrites of S1 layer 2/3 pyramidal neurons: fewer intersections in the torpor condition than in the nonhibernating condition between 10 and 90 μm (and at 110 μm) and the arousal from torpor condition between 20 and 120 μm. Length basal dendrites was shorter in the torpor condition than in the nonhibernating condition between 20 and 130 μm and the arousal from torpor condition between 20 and 120 μm. ***C***, Same as ***B*** but for basal dendrites of V1 layer 2/3 pyramidal neurons: fewer intersections in the torpor condition than in the nonhibernating condition between 10 and 70 μm and the arousal from torpor condition between 10 and 60 μm. There were fewer intersections in the arousal from torpor condition than in the nonhibernating condition at 110 μm. The length of the basal dendrites was shorter in the torpor condition than in the nonhibernating condition between 20 and 70 μm and the arousal from torpor condition between 20 and 60 μm. ***D***, Same as ***C*** but for V1 layer 2/3 pyramidal neurons collected 6 months after hibernation, with no differences across conditions. ***E***, Same as ***A*** but for V1 layer 4 spiny stellate neurons, showing no significant differences across conditions. *p < 0.01; **p < 10^−3^; ***p < 10^−4^.

In S1, both layer 4 spiny stellate neurons and layer 2/3 pyramidal neurons showed reduced dendritic arborization during torpor, which confirmed and extended previous observations (von der Ohe et al., 2006). Our quantitative analysis of the neuron morphology revealed the reduction of dendritic arborization in S1 layer 4 spiny stellate neurons during torpor, as well as a reversal within ∼1.5 h of arousal from torpor, thus confirming previous observations (von der Ohe et al., 2006). Also consistent with the previous findings, the changes were most pronounced at intermediate and higher branch orders. These findings also serve as a positive control, indicating that the lack of torpor-related changes for the V1 layer 4 spiny stellate neurons cannot be explained by methodological consequences that preferentially affect the smaller type of neuron. In addition to this control analysis, we also examined changes for an additional cell type, S1 layer 2/3 pyramidal neurons. For these, the overall dendrite length, complexity, and number of nodes were reduced in both apical and basal dendrites in the torpor group compared with the nonhibernating and arousal from torpor groups (Fig. 5*B*–*D*). When the measurements of the basal and the apical dendrites are combined, this revealed on average a 48% reduction in the number of nodes and 46% reduction in dendrite length during torpor compared with the nonhibernating group. For this cell type, the reduction in arborization was more pronounced close to the soma, as revealed by the branch order and Sholl analysis comparing the torpor and nonhibernating groups (Figs. 5*E*, 6*D*), mirroring the pattern of the V1 layer 2/3 pyramidal neuron.

## Discussion

The results here show that pronounced hibernation-related plasticity is present in V1 of 13-lined ground squirrels, at the level of layer 2/3/ pyramidal neurons, a major excitatory cell type. This plasticity expresses as a sizeable (>50%), transient reduction in dendritic arborization during torpor, the phase of deep hibernation, which rapidly and fully reverses during the intertorpor arousal epochs, without apparent long-lasting differences beyond the hibernation season. Layer 4 spiny stellate neurons, also excitatory neurons but less common in V1, showed no significant hibernation-related plasticity, contrary to layer 4 spiny stellate neurons in S1. The torpor-related plasticity in spiny stellate neurons in S1 replicates previous findings and anchors our study here to previous descriptions of torpor-related plasticity (von der Ohe et al., 2006). It is therefore unlikely that the absence of torpor-related plasticity in V1 layer 4 spiny stellate neurons reflects methodological differences between studies. Strong torpor-related plasticity was further present in layer 2/3 pyramidal neurons in S1, comparable to those in the same cell type in V1. Together, these results here both extend and complement previous work. First, our results are the first demonstration of hibernation-related plasticity in V1, making this easily accessible and relatively well-understood area a suitable target for further study of this pronounced, naturally occurring form of neuroplasticity. Second, our study revealed hibernation-related plasticity in layer 2/3 pyramidal neurons in S1, extending previous findings in this area to a new cell type. Finally, the absence of appreciable morphological changes in spiny stellate layer 4 neurons in V1, but not in S1, adds nuance to the view (von der Ohe et al., 2006) that the hibernation-related neuroplasticity is ubiquitous throughout the brain.

The hibernation-related plasticity is substantial and occurs rapidly. The dendritic arbors in V1 and S1 layer 2/3 pyramidal neurons and S1 layer 4 spiny stellate neurons reach their prehibernation levels within 1.5 h after arousal from torpor. This rapid recovery is faster than other events of dendritic remodeling such as during development. For example, V1 basal dendrites in mice grow significantly more slowly, increasing by 45% between Postnatal Day (P)7 and P23 (Richards et al., 2020). The torpor-related plasticity is also pronounced, particularly in comparison with other forms of adult neuroplasticity such as resulting from stress (Martin and Wellman, 2011) or alcohol (Staples et al., 2015). For example, stress over several days in rats has been shown to result in a reduction by ∼20% in arborization over several days in rats (Martin and Wellman, 2011). While the extent of dendritic plasticity is striking compared with other forms of neuroplasticity, our findings are comparable to the extent and speed of previous reports of torpor-related plasticity in other brain areas (von der Ohe et al., 2006). Since the previous findings were obtained using different histological approaches—iontophoretic labeling of individual neurons with Lucifer yellow followed by immunohistochemistry—it is unlikely that the pronounced torpor-related plasticity reflects a methodological artifact of the Golgi approach used here. The rapid recovery during the intertorpor arousal may partly be activity dependent. Thalamic neural activity in hibernating ground squirrels has been shown to resume as body temperature rises above 14–18°C after arousal from torpor (Krilowicz et al., 1988). Additionally, previous work proposed that the fast plasticity relates to structural protein changes rather than protein degradation and synthesis (Ruediger et al., 2007; von der Ohe et al., 2007; Schwartz et al., 2013).

Basal and apical dendrites in V1 and S1 L2/3 pyramidal neurons exhibited similar patterns of retraction and regrowth during the hibernation cycle, in contrast to dendritic remodeling observed, for example, under chronic stress. Under chronic stress, layer 2/3 pyramidal neurons’ apical dendritic trees are reduced, whereas basal dendrites remained unchanged in prefrontal and auditory cortices (Bose et al., 2010; Martin and Wellman, 2011). The more uniform plasticity observed during hibernation may result from the fluctuations in temperature that affect both apical and basal dendrites similarly.

Lack of input from the sensory periphery may contribute to the torpor-related plasticity but unlikely explains the differences between cell types. In the visual system, the lack of sensory input during torpor results from the darkness inside the burrow during hibernation and structural changes in the retina. Electron microscopy of retinas from hibernating squirrels showed a transient reduction in the cone outer segment and loss of ribbon synapses (Kuwabara, 1975; Reme and Young, 1977) during hibernation. Tracking retinas throughout hibernation in an intrasubject design using adaptive optics scanning light ophthalmoscopy showed that cone structure changes during torpor bouts transiently recovers during interbout arousal and fully recovers after hibernation (Sajdak et al., 2018).

The dramatic, rapid torpor-related plasticity documented here for the primary visual cortex lays the foundation for comparisons with other manifestations of neuroplasticity in the visual cortex. For example, ocular dominance plasticity during development has been intensely studied and is perhaps the best understood form of neuroplasticity in the cortex. Several mechanisms have been implicated in the control of this developmental neuroplasticity during the critical period (Reh et al., 2020). First, parvalbumin-positive interneurons have been shown to be important for closing and reopening the critical period. Second, extracellular matrix structure elements, such as perineuronal nets, control neuroplasticity (Sorg et al., 2016). Third, neuromodulator systems such as the cholinergic (Morishita et al., 2010) or serotonergic (Maya Vetencourt et al., 2008; Balog et al., 2014), system have further been implicated in influencing the critical period and developmental neuroplasticity.

Previous work identified body temperature as a brain-wide driver of the torpor-related neuroplasticity, but factors controlling developmental plasticity may contribute to the differences in torpor-related plasticity between cell types within V1. For example, paralleling the role of perineuronal nets in developmental plasticity, changes in perineuronal net coverage in the hypothalamus have been proposed to contribute to the control of hibernation phases (Marchand and Schwartz, 2020). Perineuronal nets coverage was reported for the dorsal cortex during hibernation, but differential expression across cell types in the visual cortex was not examined. Microglia involved in developmental plasticity in the visual cortex may further contribute to the differential hibernation-related effects (Sipeet al., 2016). Additionally, parvalbumin-positive interneurons were reduced in S1 during the winter season (Ray et al., 2020) in tree shrews. In mice, both layer 4 spiny stellate neurons in S1 and layer 2/3 pyramidal neurons in V1 and S1 form strong reciprocal connections with parvalbumin-positive interneurons (Scala et al., 2019). Assuming that the connectivity is similar in the Sciuridae, the strong connections with parvalbumin-positive interneurons are consistent with the hypothesis that parvalbumin-positive interneurons are also involved in the torpor-related plasticity in V1 and S1. In contrast, layer 4 spiny stellate neurons in V1 are sparse in mice, rats, and guinea pigs (Peters and Kara, 1985; Saez and Friedlander, 2009; Scala et al., 2019), and their connectivity with parvalbumin-positive interneurons is unclear. Differential connectivity with parvalbumin-positive interneurons would be consistent with the observed differences in their torpor-related plasticity but requires future study.

The pronounced torpor-related plasticity has translational potential (Shi et al., 2026) beyond the physiological adaptations by hibernators to tolerate hypothermia (Ou et al., 2018) and metabolic stress (Dave et al., 2012). Indeed, insights from natural torpor are sought to be leveraged for clinical conditions ranging from neurodegeneration to stroke, as well as nonclinical applications such as manned spaceflight (Shi et al., 2026). In conjunction with the broad knowledge on synaptic plasticity during development (Reh et al., 2020), torpor-related plasticity in the visual cortex promises to be a particularly fruitful platform.
